# Personal beliefs and social norms regarding the sexual exploitation of girls in age-disparate transactional sexual relationships in Brazil: a mixed-methods study

**DOI:** 10.1186/s12978-022-01437-3

**Published:** 2022-06-06

**Authors:** Caroline Ferraz Ignacio, Linda Cerdeira, Beniamino Cislaghi, Giovanna Lauro, Ana Maria Buller

**Affiliations:** 1 Promundo-US, 1367 Connecticut Avenue NW, Suite 310, Washington, DC 20036 USA; 2 Promundo Portugal, Centro de Estudos Sociais/ Universidade de CoimbraColégio de S. Jerónimo, Largo D. Dinis, Apartado 3087, 3000-995 Coimbra, Portugal; 3 Department of Global Health and Development, London School of Hygiene and Tropical Medicine, Keppel Street, London, WC1E7HT UK

**Keywords:** Youth, Sex, Relationships, Gender norms, Urban health, Masculinity, Brazil, Latin America

## Abstract

**Background:**

In the global debate around transactional sex little attention has concentrated on Brazil, despite ranking fourth globally in absolute number of girls married or co-habiting by the age of 15 years, and evidence showing that these unions often begin as age-disparate transactional sex (ADTS). This article contributes to filling this gap by exploring the personal beliefs and social norms related to ADTS in urban (favela) communities of Rio de Janeiro, Brazil between adult men (> 18 years) and girls and adolescents (G/A) (< 18 years) with a minimum 5-year age disparity. The primary objective of this study was to identify the social norms that promote and prevent ADTS, and the dynamics between individual beliefs and social norms, to provide contextualized recommendations to prevent ADTS in this setting.

**Methods:**

An exploratory, sequential, mixed-methods design was used, starting with a qualitative phase that included semi-structured, in-depth interviews and focus groups, and a subsequent quantitative phase comprising of a community survey. The items for the quantitative questionnaires were developed based on the qualitative results.

**Results:**

Mixed methods results indicate that in these communities ADTS is normalised and not considered exploitative. We identified three themes related to the reasons ADTS occurs: girls’ responsibility, male desires and benefits of ADTS. Men’s role in ADTS was largely minimised because of a general acceptance of a notion of masculinity characterised by hypersexuality and lack of impulse control. Individual beliefs, however, did not tend to align with these social norms.

**Conclusions:**

In this study, personal beliefs and social norms often did not align, suggesting that initiatives working to change personal or attitudes regarding ADTS may not lead to meaningful change in ADTS behaviours, and social norms interventions may be more effective. Our findings reinforce the need to develop programs tailored to local understandings of ADTS, targeting not only girls but also a wide range of actors. Interventions could also consider the structural factors acting in local and global contexts that promote or prevent ADTS.

## Plain language summary

This article explores the personal beliefs and social norms related to the exchange of sexual favours or relationships for material favours, gifts and/or support in some form, between adult men (> 18 years) and girls and adolescents (< 18 years) with a minimum 5-year age difference. We used interviews, focus groups and questionnaires to understand the factors that promote and prevent these sexual relationships between men and girls. Motivators for these relationships were often related to girls’ responsibilities, male desires and the benefits of these relationships. Men’s responsibility for their participation in these relationships with girls were often minimised due to a general acceptance of men as overly sexual and lacking impulse control. In this study, personal beliefs and social norms were often not aligned, suggesting that interventions focused on changing personal beliefs or attitudes about these sexual relationships may not be enough to change social norms. The findings highlight the need to develop solutions that consider a wider range of actors, instead of interventions focused only on girls. The study findings also support the need to further investigate how communities and shared expectations can influence sexual relationships in exchange for goods between adult men and girls and adolescents.

## Background

The sexual exploitation of children and adolescents (SECA) includes a variety of behaviours such as exposing children to pornography, sexual touching, online solicitations of pictures or sexual material, trafficking, child marriage and/or transactional sex. Whereas commercial forms of SECA and paedophilia have been visibly and unequivocally condemned by communities, non-commercial transactional sex has received less attention, except in Sub-Saharan Africa where it has been identified as a driver of the HIV epidemic and attracted research attention (Muthoni et al., 2020)[16]. There is also less consensus on the classification of transactional sex as SECA [10, 12, 18, 25–27]. A lack of a clear conceptualisation of transactional sex, combined with the sensitive nature of this behaviour and associated barriers to reporting, have led to underestimates of its prevalence [10].

In 1990, Brazil was one of the first countries to develop a legal framework specifically focused on children and adolescents (up to 18 years)—The Child and Adolescent Statute—inspiring legal reforms throughout Latin America [6]. Since then, the Brazilian government has created the National Programme to Confront Sexual Violence Against Children and Adolescents, an intersectoral commission and continued legislation focused on protecting against SECA.

Despite an advanced legal framework for protecting children, estimates indicate that in Brazil 36% of women aged 20–24 years were married or cohabiting before age 18, and 11% by age 15 [23]. In absolute numbers, Brazil occupies the fourth global position in number of girls married or co-habiting by 15 years-old. Yet, Brazil receives little attention in the global debate around child marriage. Previous research on child marriage in Brazil has shown that these unions often begin as transactional sex, which has been defined as the exchange of sexual favours or relationships for benefits such as material favours, gifts and/or support in some form [22].

The present study concentrates on age-disparate transactional sex (ADTS), defined as transactional sex between girls less than 18 years-old and adult men at least five years older [13]. This study builds on research that aims to understand the social norms that prevent or promote SECA with the goal of bolstering primary prevention efforts. There are a variety of social norms theory approaches [14], but the most commonly used in global health, and the one we adopt for this study, is that by Cialdini and colleagues [4] who defines norms as one’s beliefs about what others do and what others approve of. According to this definition norms can act as breaks for change even when people’s own personal beliefs and attitudes (internal disposition towards something) are aligned with the desired behaviour. Hence, interventions that aim to exclusively changing people’s attitudes towards a given practice may be insufficient for changing practices in communities. Our study aims to contribute to the existing literature and provide contextualised recommendations to prevent ADTS in Brazil by shedding light on the kind of social norms that support or prevent ADTS, and estimate the prevalence and dynamics of ADTS in Brazil.

## Methods

This exploratory sequential mixed-methods study [5] consisted first of a formative, qualitative phase to identify social norms and personal beliefs related to ADTS, which then informed the development of personal beliefs and social norms items for a quantitative survey. This survey was then applied in the quantitative phase to test the distribution of the items in the population and their associations with socio-demographic characteristics and participation in ADTS.

### Study setting

The study was conducted in informal urban, low-income communities called *favelas*, in the city of Rio de Janeiro, Brazil.

The qualitative phase was conducted in three different favelas within Rio de Janeiro: *Cidade de Deus* in the western zone of the city, *Rocinha* in the southern zone *and Nova Hollande* in the northern zone. These communities were selected because of their high level of vulnerability according to their score on the Social Vulnerability Index (SVI) (IPEA, http://ivs.ipea.gov.br/index.php/pt/). The SVI considers dimensions related to urban infrastructure, human capital and income and labour (for more details see [10].

We had planned to conduct the quantitative phase in two of the same communities in which we had conducted the qualitative phase, however, due to security concerns arising from armed conflict in *Cidade de Deus* and *Rocinha* during qualitative fieldwork, this was not possible. After careful consideration and guided by location, similar characteristics and SVI scores we chose *Babilônia* /*Chapéu* *Mangueira* in the southern zone of the city as an alternative favela for the quantitative phase of the study instead of Rocinha. The decision to enter any community was taken following the research teams’ and community leaders’ judgment about the safety conditions on the day, relying on the following criteria: (1) the presence of military police, (2) recent or active gunfire, (3) closed commerce during normal business hours, (4) rumours of an impending military police invasion, and/or (5) rumours of an arrival of a large drug shipment.

The questionnaires for the quantitative phase were pre-tested in *Cidade de Deus*.

### Qualitative data collection and analysis

We partnered with community leaders and resident associations to identify men and women residents from 15 to 65 years using non-probability purposive sampling. In-depth interviews and focus groups were conducted with a total of 130 participants sampled by gender and community. We conducted 30 semi-structured, in-depth interviews and 10 focus groups in total with a range of 8 to 12 participants in each focus group. Interviews took between 45 and 60 min and focus group discussion between 60 and 90 min. Fieldwork was conducted between June and August 2017. Overall, there were 130 participants in the qualitative phase: 35 aged 15–17 years, 30 aged 18–24 years, 16 aged 25–34 years, 32 aged 35 to 45 years, and 27 aged 46–65 years (Tables 1 and 2). All interviews and focus groups were audio recorded.

**Table 1:** Characteristics of the interview participants

Age group	Community (boys and men interviewees / girls and women interviewees)			Total interviews per age group
	RO	CM	CDD	
15–17	1 / 1	1 / 1	0 / 1	5
18–24	1 / 1	2 / 1	3 / 2	10
25–34	1 / 1	0 / 2	1 / 1	6
35–44	1 / 1	0 / 0	0 / 0	2
46–65	1 / 1	1 / 2	1 / 1	7
Total interviews per community	10	10	10	30 total interviews

Source: Ignacio et al. [10]

**Table 2:** Characteristics of the focus group participants

Age group	Community (boys and men groups / girls and women groups)			Total focus groups per age group
	RO	CM	CDD	
15–17	0 / 1	1 / 0	1 / 0	3
18–24	0 / 0	1 / 0	0 / 1	2
25–34	0 / 0	0 / 0	0 / 1	1
35–45	0 /1	1/ 0	1/0	3
46–65	1/ 1	0 / 0	0 / 0	2
Total focus groups per community	4	3	4	10 total focus groups

Source: Ignacio et al. [10]

The number of interviews was decided pragmatically to allow for an equal number with women and men and an equal distribution among the study sites. Analysis started during fieldwork and the team established that saturation was reached within 30 interviews.

During the interviews and focus groups we used vignettes specifically designed to elicit personal beliefs and social norms around ADTS. The vignettes were created based on the literature regarding ADTS, meetings with key stakeholders, and consultation with experts. The interviews and focus groups’ topic guides included the same questions addressing: personal beliefs and community social norms related to SECA; patterns of sexual exploitation in favelas in Brazil; social norms that protect against or foster ADTS; if/how ADTS is accepted or sanctioned in the communities (Ignacio et al. 2019). Data collection was conducted in Portuguese by local experienced researchers. Qualitative instruments were pilot tested with groups of the same demographic profile as the target population but from different low-income neighbourhoods to assess relevance of the vignettes and acceptability of the length of the interviews.

Transcripts and field notes were anonymised. A thematic analysis approach was used to identify themes related to the social norms that promote and protect against ADTS. Four members of the research team identified thematic codes, sub-themes and exemplifying quotes. The codes from the different team members were discussed and merged. Transcripts were coded manually. For more detailed information regarding the qualitative analysis is available in Ignacio, et al. [10].

### Quantitative data collection and analysis

To develop the quantitative data collection tool the team held multiple face-to-face and virtual meetings with experts from Promundo-US as well as the London School of Hygiene and Tropical Medicine (LSHTM) and two local statisticians. Items were developed based on the qualitative results with domains and items selected to reflect those personal beliefs and social norms most prevalent in the qualitative data. The team then developed two sets of introductory statements and response scales. The social norms items started with “How many people in your community think that….” and had the answer options: “everyone”, “the majority”, “half”, “a few”, and “no one”. The personal belief items started with “Do you think that…” and had the following answer options: “totally agree”, “agree”, “neither agree nor disagree”, “disagree”, and “totally disagree”. The items were evaluated based on their clarity, relevance and importance by a panel of seven external experts, then pretested in CDD. From the pre-test data, 41 items were selected to be included in the final questionnaire. Additionally, three questions regarding respondents’ practices were included: if the respondent had ever had sexual relations in exchange for gifts or other material goods; if the respondent had sexual relations with an adult while still a minor; and if the adult respondents had had sexual relations with minors while adults.

412 participants were selected using a multi-stage sampling plan (census sectors, households, resident). Eight census tracts or sectors were randomly selected from each community. The households in each sector were identified and numbered. A number representing a household was randomly selected as the starting point in the sector. For each sector, data collection teams were given sector maps, a description of the sector and a table of quotas according to sex (50% male and 50% female) and age of residents (with a 1/3 of the sample in each of the age groups (15–17, 18–24, and 25 + years) needed for that sector. After household selection, the interviewer team requested a resident that met the sector quota guideline age and gender.

Questionnaires were conducted from the 27th July to the 2nd November, 2018 in the homes of the participants using tablets with the software CommCare by Dimagi. Interviewers were gender-matched with participants. The final sample included a total of 412 residents of the study communities: 280 (68.0%) from NH and 132 (32.0%) from B/CM. The socio-demographic characteristics of the participants is available in Table 3.

**Table 3:** Individual characteristics of the participants

Characteristic	Total n (%)	NH	B/CM	*p*-value
Gender				0.34
Female	220 (53.4)	145 (51.8)	75 (56.8)	
Male	192 (46.6)	135 (48.2)	57 (43.2)	
Age group				** < 0.001**
Adolescent (15–17 years)	129 (31.3)	109 (38.9)	20 (15.1)	
Young adult (18–29 years)	169 (41.0)	115 (41.1)	54 (40.9)	
Adult (30–44 years)	63 (15.3)	30 (10.7)	33 (25.0)	
Middle-aged adult (24–59 years)	40 (9.7)	23 (8.2)	17 (12.9)	
Seniors (≥ 60 years)	11 (2.7)	3 (1.1)	8 (6.1)	
Status in the home in relation to head of household				** < 0.001**
Head of household (HOH)	159 (38.6)	79 (28.2)	80 (60.6)	
Partner	32 (7.8)	27 (9.6)	5 (3.8)	
Child or step-child	183 (44.4)	140 (50.0)	43 (32.6)	
Parent or parent-in-law	6 (1.5)	6 (2.1)	0	
Grandchild or great-grandchild	18 (4.4)	16 (5.7)	2 (1.5)	
Sibling	5 (1.2)	5 (1.8)	0	
Other relative	9 (2.2)	7 (2.5)	2 (1.5)	
Educational attainment				** < 0.001**
No formal education	5 (1.2)	5 (1.8)	0	
Complete elementary school	23 (5.6)	16 (5.7)	7 95.3)	
Incomplete middle school	97 (23.5)	76 (27.1)	21 (15.9)	
Complete middle school	65 (15.8)	50 (17.9)	15 (11.4)	
Incomplete high school	100 (24.3)	69 (24.6)	31 (23.5)	
Complete high school	72 (17.5)	38 (13.6)	34 (25.8)	
Higher level education	1 (0.24)	0	1 (0.8)	
Currently studying				0.04
Yes	147 (35.7)	109 (38.9)	38 (28.8)	
No	265 (64.32)	171 (61.1)	94 (71.2)	
Economically active during the last month				** < 0.001**
Yes	160 (38.8)	89 (31.8)	71 (53.8)	
No	241 (58.5)	180 (64.3)	61 (46.2)	
Never worked	11 (2.7)	11 (3.9)	0	
Religion				0.02
Catholic	111 (26.9)	72 (25.7)	39 (29.5)	
Protestant, Pentecostal, or Evangelical	153 (37.1)	97 (34.6)	56 (42.4)	
Spiritism or Kardecist	4 (1.0)	3 (1.1)	1 (0.8)	
Umbanda or Candomblé (African origin)	9 (2.2)	3 (1.1)	6 (4.6)	
No religion	134 (32.5)	104 (37.1)	30 (22.7)	
Unanswered	1 (0.2)	1 (0.4)	0	
Importance of religious beliefs				0.02
Very important	131 (31.8)	77 (27.5)	54 (40.9)	
Important	114 (27.7)	75 (26.8)	39 (29.6)	
A little important	24 (5.8)	16 (5.7)	8 (6.1)	
Not important	5 (1.2)	5 (1.8)	0	
Unanswered	7 (1.7)	6 (2.1)	1 (0.8)	
Not applicable	131 (31.8)	101 (36.1)	30 (22.7)	

Questionnaire data were exported to excel and analysed using Epi Info 7. Bivariate and multivariate analyses were conducted using gender, age range, community of residence, poverty level, and importance of religious beliefs variables. The association between these variables and each personal belief and social norm item was tested. Associations between the questions about behaviours and those about gender, age range, community of residence, poverty level, and importance of religious beliefs were analysed. Associations between the items were also analysed. At the final stage of analysis we integrated the mixed-methods data by triangulating the qualitative and quantitative findings, and interpreting and discussing them together.

## Results

Four main themes arose from our convergent analysis of both datasets. The first described the prevalence and experience of ADTS in the context of the favelas, where there are high rates of drug trafficking activity. The second theme related to the role of the family in ADTS, with a particular focus on mothers and friends as important reference groups for this practice. The third and fourth themes were related to the responsibilities and motivations of men and girls to engage in ADTS. When describing each theme we draw on both qualitative and quantitative data.

### Prevalence and experience of ADTS

Overall, 45.9% of all participants believed that it was normal for men to get involved in sexual relationships with girls and 48.3% expected their community members to also see the behaviour as normal. Among these, adolescents and young adults (independent of their sex) were the most likely to normalize ADTS relationships. Conversely, those with strong religious beliefs were less likely than those with weaker religious beliefs to accept it as normal, although even within the latter group the majority thought that most or all of their community members would support ADTS (43.7% versus 55.1%, p-value = 0.021). Adults who considered their religion important were also less likely to have had sexual relationships with a minor (OR: 0.447, 95%CI: 0.233–0.859, p-value: 0.012) (See Table 4).

**Table 4:** Self-reported respondent practices related to ADTS

Characteristic	Men, n (%)	Women, n (%)	p-value	Total n (%)
Has had sexual relations in exchange for gifts or material goods (n = 412)			** < 0.001**	
Yes	48 (25.0)	17 (7.7)		65 (15.8)
No	136 (70.8)	203 (92.3)		339 (82.3)
Unanswered	8 (4.2)	0 (0.0)		8 (1.9)
As a minor, has had sexual relations with an adult (n = 412)			** < 0.001**	
Yes	94 (49.0)	87 (39.6)		181 (43.9)
No	90 (46.9)	133 (60.5)		223 (54.1)
Unanswered	8 (1.9)	0 (0.0)		8 (2.0)
As an adult, has had sexual relations with a minor (n = 283)			** < 0.001**	
Yes	36 (27.3)	8 (5.3)		44 (15.6)
No	90 (68.2)	143 (94.7)		233 (82.3)
Unanswered	0 (0.0)	6 (4.5)		6 (2.1)

Our results suggest that men involved in drug trafficking were expected to also be involved in ADTS either as perpetrators engaging in ADTS or as the only members of the community who were capable of interfering to end these relationships.

When asked about how much girls were attracted to men involved in drug trafficking, women were significantly more likely to disagree that girls were attracted to them than men. The qualitative results provided more nuance to these results. Participants narratives confirmed that drug dealers’ symbolic and economic power was appealing to girls, but they also revealed that this same power created an environment of fear among community members, who were conditioned to not question or even discuss practices related to drug trafficking—including not interfering in girls’ ADTS relationships with traffickers. Fear seemed to limit girls’ ability to leave or refuse entering into an ADTS relationship with a man, even more so if the man was connected to drug trafficking.

“She has to be decent, nowadays the way you see a child on this side is not the same way you see the girls on the other side of the alley [where drugs were sold or consumed], the child from this side is quieter, she stays more at home, the girl from the other side doesn’t do that, she stays on the street all the time, she goes to the beach, she’s all about the beach, the rooftop, if it’s sunny, she goes to the roof slab. How come ain’t nobody going to flirt with her? It’s hard. No one can say ‘I won’t do it’.” (Man, 32, CDD, interview)

Men involved in drug trafficking were the only actors in the favela allowed to interfere in other people’s relationships. They were hence seen as protectors: girls or their families could ask for their help when they wanted to end a relationship, but also felt afraid of a violent retaliation. Furthermore, residents believed that drug traffickers could punish men involved in ADTS if they saw the relationship as inappropriate, regardless if the family or the girl had asked the drug traffickers for help.

“This story of seeing men, paedophiles, an older man hooking up with a child, that’s happened here, but they killed him. The law here is the law of the streets, do you understand?” (Woman, 53, CM, interview)

Participants believed that drug traffickers’ disapproval would increase as girls’ age decreased: the younger the girl, the greater the likelihood of traffickers interfering (Tables 5 and 6). It should be stressed that the role of drug trafficking in each community is complex and its role in promoting or preventing ADTS was not consistent among communities and/or participants, especially in terms of what drug traffickers would consider ‘inappropriate’ enough to warrant interference.

**Table 5:** Personal attitudes regarding drug trafficking and ADTS

	Response				Age group	Gender
Phrases	Agree or totally agree	Don’t agree nor disagree	Disagree or totally disagree	No answer	Df = 12	Df = 5
You think…	*n* (%)	*n* (%)	*n* (%)	*n* (%)	*x*^2^, *p-*value	*x*^2^, *p-*value
A girl can refuse to get involved with a trafficker that wants to be with her	311 (75.5)	35 (8.5)	60 (14.6)	6 (1.5)	13.328, 0.346	6.920, 0.074
Families of the girls involved with men can ask for help from the traffickers	134 (32.5)	62 (15.0)	191 (46.4)	25 (6.1)	13.414, 0.340	2.544, 0.467
Men who get involved with girls of 12 years old or younger will be punished by traffickers because of this relationship	184 (44.7)	60 (14.6)	133 (32.3)	35 (8.5)	17.710, 0.125	4.222, 0.243
Men who get involved with girls of 13 to 14 years old will be punished by traffickers because of this relationship	148 (35.9)	75 (18.2)	154 (37.4)	35 (8.5)	11.919, 0.452	4.175, 0.243
Men who get involved with girls of 15 years old or older will be punished by traffickers because of this relationship	76 (18.5)	74 (18.0)	233 (56.5)	29 (7.0)	18.275, 0.108	3.392, 0.335
Girls like men involved in drug trafficking because they are able to financially support girls	267 (64.8)	66 (16.0)	63 (15.3)	16 (3.9)	14.347, 0.279	**11.887, 0.008**
Girls like men involved with trafficking because they are powerful in the community	298 (72.3)	57 (13.8)	42 (10.2)	15 (3.6)	9.582, 0.653	**10.124, 0.018**
Girls that are involved with men in drug trafficking have a superior status	222 (53.9)	72 (17.5)	99 (24.0)	19 (4.6)	16.772, 0.158	**14.540, < 0.001**

**Table 6:** Perceptions of community beliefs regarding drug trafficking and ADTS

	Responses				Age group	Gender
Phrases	Most people or everyone	Half	Few or no one	No answer	Df = 12	Df = 5
How many people in your community believe that…	*n* (%)	*n* (%)	*n* (%)	*n* (%)	*x*^2^, *p-*value	*x*^2^, *p-*value
A girl can refuse to get involved with a trafficker that wants to be with her	227 (55.10)	60 (14.6)	98 (23.8)	27 (6.5)	11.111, 0.519	**15.832, < 0.001**
Families of the girls involved with men can ask for help from the traffickers	151 (36.7)	73 (17.7)	146 (35.4)	42 (10.2)	12.519, 0.405	5.025, 0.170
Men who get involved with girls of 12 years old or younger will be punished by traffickers because of this relationship	133 (32.3)	79 (19.2)	150 (36.4)	50 (12.1)	10.125, 0.605	7.223, 0.065
Men who get involved with girls of 13 to 14 years old will be punished by traffickers because of this relationship	117 (28.4)	72 (17.5)	172 (41.7)	51 (12.4)	9.513, 0.659	4.658, 0.199
Men who get involved with girls of 15 years old or older will be punished by traffickers because of this relationship	65 (15.8)	60 (14.6)	234 (56.8)	53 (12.9)	10.896, 0.538	4.933, 0.177
Girls like men involved in drug trafficking because they are able to financially support girls	237 (57.5)	57 (13.8)	85 (20.6)	33 (8.0)	13.693, 0.321	4.504, 0.212
Girls like men involved with trafficking because they are powerful in the community	259 (62.9)	49 (11.9)	75 (18.2)	29 (7.0)	11.627, 0.476	6.124, 0.106
Girls that are involved with men in drug trafficking have a superior status	223 (54.1)	64 (15.5)	95 (23.1)	30 (7.3)	14.402, 0.276	5.737, 0.125

### The role of the family and friends in ADTS

Within the family, mothers were exclusively held responsible for their daughters’ involvement in ADTS, with most women assuming the role of sexual “educators” and “protectors” of girls to conform with what they perceived to be the expectations of other women in their community.

“The guidance has to come from the parents, (…) that means, the mother has to sit, talk, explain to the daughter how things are.” (Woman, 35-44, RO, Focus Group)

The quantitative data confirmed the importance of the family’s role and men were likely to both believe and expect their communities to agree that the family is responsible for the sexual behaviour of a girl (Tables 7 and 8).

**Table 7:** Personal attitudes regarding the role of family and friends

	Response				Age group	Gender
Phrases	Agree or totally agree	Don’t agree nor disagree	Disagree or totally disagree	No answer	Df = 12	Df = 5
You think…	*n* (%)	*n* (%)	*n* (%)	*n* (%)	*x*^2^, *p-*value	*x*^2^, *p-*value
The family is responsible for the sexual behavior of a girl	219 (53.2)	64 (15.5)	125 (30.3)	4 (1.0)	10.950, 0.533	**27.711, < 0.001**
The family is judged by the girl's sexual behavior	254 (61.6)	65 (15.8)	91 (22.1)	2 (0.5)	8.155, 0.773	2.498, 0.476
Girls that get involved with men are seen as popular by other girls	202 (49.0)	51 (12.4)	153 (37.1)	6 (1.5)	10.249, 0.594	**20.609, < 0.001**
Female friends encourage each other to have relationships with men	303 (73.5)	45 (10.9)	62 (15.1)	2 (0.5)	12.067, 0.440	25.012, < 0.001

**Table 8:** Perceptions of community beliefs regarding the role of family and friends

	Responses				Age group	Gender
Phrases	Most people or everyone	Half	Few or no one	No answer	Df = 12	Df = 5
How many people in your community believe that…	*n* (%)	*n* (%)	*n* (%)	*n* (%)	*x*^2^, *p-*value	*x*^2^, *p-*value
A family is responsible for the sexual behavior of a girl	216 (52.4)	88 (21.4)	95 (23.1)	13 (3.2)	17.167, 0.143	**8.403, 0.038**
The family is judged by the girl's sexual behavior	227 (55.1)	95 (23.0)	74 (18.0)	16 (3.9)	**20.832, 0.053**	4.758, 0.190
Girls that get involved with men are seen as popular by other girls	190 (46.1)	102 (24.7)	107 (26.0)	13 (3.2)	17.381, 0.136	**10.120, 0.018**
Female friends encourage each other to have relationships with men	276 (67.0)	56 (13.6)	67 (16.2)	13 (3.2)	**26.017, 0.011**	**19.000, < 0.001**

In relation to the link between popularity and ADTS, quantitative data show that the majority of men believed that girls who get involved with men are seen as popular by other girls and expected their community members to agree (59.9% and 54.2%, respectively). Tables 5 and 6 show responses of all participants not disaggregated by gender or age. Most men and women agreed that female friends encouraged each other to have relationships with men (83.4% of men and 65.0% of women); but women were significantly more likely to disagree. However, girls did not agree with the idea that their friends encouraged ADTS, highlighting differences in beliefs between life stages.

For the men, their male friends and the women of the community were important reference groups to whom men were expected to ‘prove’ their masculinity. Hypersexuality, exhibited by “conquering” young girls and having multiple sexual partners, was seen as a way of affirming themselves as “real men”.

“I think that for them it’s an issue of masculinity: I did it, I conquered one more, conquered her, the young one. So, I think that’s it, I think it’s his ego.” (Woman, 33, CM, interview)

“Boys have a gigantic influence, for you to be a man you can’t be a virgin. You have to hook up with lots of girls.” (Woman, 27, CM, interview)

### Men’s responsibilities and motivations for ADTS

During the qualitative phase, participants normalized men’s participation in ADTS as “normal” and expected [10]. The quantitative findings supported this and Tables 7 and 8 show participant responses regarding men’s motivations for ADTS.

Seniors (72.7% [8/11]) and adolescents (65.1% [84/129]) were more likely to believe that men never refuse sex offered by a girl (p-value < 0.001). Adolescents were more likely than seniors to believe that their communities expect men to never refuse sex offered by a girl (62.0% [80/129]; p-value = 0.012).

Middle-aged adults were more likely to totally disagree (17.5% [7/40]) and seniors to agree (54.6% [6/11]) that “men get more pleasure from sex with girls than with women” (p-value < 0.001).

Middle-aged respondents (p-value = 0.045) and men (p-value < 0.001) were more likely to agree that girls were manipulating the men they were involved with. Specifically, men believed more frequently that even though men benefit more from sexual relationships with girls, girls gain financial stability through involvement with adult men (45.3% of men versus 25.5% of women) (p-value < 0.001).

Despite residing in the same communities, the results show that the participants did not all interpret the social norms regarding girl’s perceived manipulation of men in the same way. Women were less likely than men to perceive community agreement that adult men manipulate their underage female partners (p-value < 0.001 in logistic regression). Men were divided on whether they expected their communities to believe that girls who are involved with men are manipulating them: 35.9% [69/192] of men expected the communities to be supportive of this belief. Conversely, women more frequently believed that their community had unified positions: 49.6% [109/220] of women expected their communities to not support the view of girls as the manipulators (p-value < 0.001, logistic regression). Adolescents were less likely to believe that their community members agreed that men benefit more (p-value = 0.054) (Tables 9 and 10).

**Table 9:** Personal attitudes regarding men's motivations for ADTS

	Response				Age group	Gender
Phrases	Agree	Don’t agree nor disagree	Disagree	No answer	Df = 12	Df = 5
You think…	*n* (%)	*n* (%)	*n* (%)	*n* (%)	*x*^2^, *p-*value	*x*^2^, *p-*value
Men get involved with girls because they cannot control their sexual desires	193 (46.8)	61 (14.8)	143 (34.7)	15 (3.6)	**22.882, 0.029**	7.120, 0.068
Men think that the body of a girl is more attractive than that of a woman	247 (60.0)	64 (15.5)	92 (22.3)	9 (2.2)	**22.588, 0.031**	0.694, 0.875
Men like to get involved with girls because they are easier to control than women	276 (67.0)	47 (11.4)	82 (19.9)	7 (1.7)	14.268, 0.284	1.105, 0.776
Men never refuse sex offered by a girl	222 (53.9)	69 (16.7)	107 (26.0)	14 (3.4)	**32.070, < 0.001**	0.309, 0.958
Men get more pleasure from sex with girls than with women	155 (37.6)	84 (20.4)	146 (35.4)	27 (6.6)	**39.247 < 0.001**	1.642, 0.650
Men feel more powerful in sexual relations with girls than with women	208 (50.5)	60 (14.6)	124 (30.1)	20 (4.9)	**23.810, 0.022**	4.911, 0.178
If a man gets involved with a girl, he's seen as virile by women in the community	148 (35.9)	59 (14.3)	196 (47.6)	9 (2.2)	**21.941, 0.038**	1.154, 0.764
Girls that are involved with men are manipulating them (the men)	123 (29.8)	94 (22.8)	189 (45.9)	6 (1.5)	**21.406, 0.045**	**23.504, < 0.001**
Men involved with girls are manipulating them (the girls)	198 (48.1)	92 (22.3)	119 (28.9)	3 (0.7)	**23.160, 0.026**	4.345, 0.227
When a girl and a man have sexual relations, the man benefits more	216 (52.4)	79 (19.2)	100 (24.3)	17 (4.1)	12.353, 0.418	5.686, 0.128

**Table 10:** Perceptions of community beliefs regarding men's motivations for ADTS

	Responses				Age group	Gender
Phrases	Most people or everyone	Half	Few or no one	No answer	Df = 12	Df = 5
How many people in your community believe that…	*n* (%)	*n* (%)	*n* (%)	*n* (%)	*x*^2^, *p-*value	*x*^2^, *p-*value
Men get involved with girls because they cannot control their sexual desires	190 (46.1)	65 (15.8)	131 (31.8)	26 (6.3)	16.719, 0.161	3.809, 0.283
Men think that the body of a girl is more attractive than that of a woman	215 (52.2)	87 (21.1)	92 (22.3)	18 (4.4)	**23.338, 0.025**	3.162, 0.367
Men like to get involved with girls because they are easier to control than women	223 (54.1)	81 (19.7)	87 (21.1)	21 (5.1)	**21.378, 0.045**	1.100, 0.778
Men never refuse sex offered by a girl	219 (53.2)	77 (18.7)	96 (23.3)	20 (4.6)	**25.578, 0.012**	3.048, 0.384
Men get more pleasure from sex with girls than with women	176 (42.7)	83 (20.2)	126 (30.6)	27 (6.5)	11.595, 0.479	3.044, 0.385
Men feel more powerful in sexual relations with girls than with women	190 (46.1)	90 (21.8)	110 (26.7)	22 (5.3)	20.527, 0.058	0.431, 0.934
If a man gets involved with a girl, he's seen as virile by women in the community	155 (37.6)	80 (19.4)	148 (35.9)	29 (7.0)	9.108, 0.694	1.610, 0.657
Girls that are involved with men are manipulating them (the men)	125 (30.3)	82 (19.9)	178 (43.2)	27 (6.6)	15.078, 0.237	**8.861, 0.031**
Men involved with girls are manipulating them (the girls)	189 (45.9)	84 (20.4)	120 (29.1)	19 (4.6)	16.434, 0.172	**8.663, 0.034**
When a girl and a man have sexual relations, the man benefits more	178 (43.2)	93 (22.6)	109 (26.5)	32 (7.8)	**20.764, 0.054**	3.796, 0.284

### Girls’ responsibilities and motivations for ADTS

As shown in previous sections, in general, and specifically among men, girls (and their families) were expected to be responsible for the occurrence of these relationships. Men were more likely to agree that a girl with a developed body is ready to have sex (p-value < 0.001); but, women (p-value = 0.006) were more likely to expect their communities to believe that a girl with a developed body has the maturity to make decisions about her sexual partners and relations. Adolescents and respondents without important religious beliefs, 27.9% (p-value < 0.001) and 25.2% (p-value < 0.001), respectively, were more likely to believe that a girl with a developed body has the maturity to make decisions about her sexual partners and relations.

Men more frequently reported that girls’ presence in the streets was a signal of her availability for ADTS (44.8% of men versus 23.6% of women) and 51.6% of men expected most of their community members to agree. Women were much more divided regarding their perceptions of their communities’ social norms: whereas 40.0% believed that at least most of the people in their communities would agree, 39.1% believed that few or no one in the community would agree with the statement regarding girls’ presence in the street.

Men more frequently believed that if a girl responds positively to the advances of a man on the street, or if she accepts presents or protection from a man, then she should have sex with him. In both communities, more residents expected their community members to disagree that these conditions should be repaid with sex, but residents of NH were more likely to expect their community members to agree (p-value < 0.001).

Among men, 64.0% believed that girls who wear short skirts are looking for male attention, compared to 18.6% of women, and 71.9% of men expected that at least half of the people in their community would agree. Differences between communities were also significant.

In terms of the motivators for girls to participate in ADTS, during the qualitative research financial interests as a motivator for girls’ participation in ADTS was not emphasized by the participants.

Similarly, in the quantitative phase, less than half of the respondents believed that men must provide financially for girls they have sexual relations with, with women more likely to disagree. Middle-aged adults were most likely to be unsure or not answer if men must provide financially for the girls they have sexual relations with (10.0% [4/40]), but adolescents and young adults were the most likely to agree with the statement, 49.6% [64/129] and 58.7% [63/169], respectively.

When considering structural drivers of ADTS, adolescents and young adults were more likely to disagree (20.5% [61/298]) that relationships between men and girls are less common when good public services exist in the community, while the majority of adults and middle-aged adults agreed with the statement (72.8% [75/103]). Respondents with important religious beliefs and men were more likely to expect their communities to agree that ADTS is less common when there are good public services (p-value = 0.014) (Tables 11 and 12).

**Table 11:** Personal attitudes regarding girls’ responsibilities and motivations for ATS

	Response				Age group	Gender
Phrases	Agree	Don’t agree nor disagree	Disagree	No answer	Df = 20	Df = 5
You think…	n	n (%)	n	n (%)	*x*^2^, p-value	*x*^2^, p-value
A girl with a developed body is ready to have sex	85 (20.6)	62 (15.0)	258 (62.6)	7 (1.7)	20.943, 0.401	**33.639, < 0.001**
A girl with a developed body has the maturity to make decisions about her sexual partners and relations	80 (19.4)	67 (16.3)	258 (62.6)	7 (1.7)	**46.948, < 0.001**	**12.249, 0.032**
A girl can refuse to get involved with a man that wants to be with her	344 (83.5)	24 (5.8)	38 (9.2)	6 (1.5)	19.962, 0.460	5.900, 0.316
If a girl responds positively to the advances of a man on the street, she should have sex with him	80 (19.4)	46 (11.2)	279 (67.7)	7 (1.7)	28.991, 0.088	**31.485, < 0.001**
Girls that spend a lot of time in the street are available to get involved with men	138 (33.5)	47 (11.4)	221 (53.6)	6 (1.5)	**32.743, 0.036**	**42.853, < 0.001**
Girls that use short skirts are looking for male attention	143 (34.7)	55 (13.3)	210 (51.0)	4 (1.0)	22.809, 0.298	**35.410, < 0.001**
Girls that accept presents or protection from men should repay with sex	66 (16.0)	31 (7.5)	309 (75.0)	6 (1.5)	23.511, 0.264	**52.456, < 0.001**
Girls of 12 years old or younger are able to choose their sexual partners and relationships	50 (12.1)	23 (5.6)	337 (81.8)	2 (0.5)	18.168, 0.576	9.326, 0.097
Girls of 13 to 14 years old are able to choose their sexual partners and relationships	78 (18.9)	39 (9.5)	293 (71.1)	2 (0.5)	20.979, 0.398	9.229, 0.100
Girls of 15 years old or older are able to choose their sexual partners and relationships	225 (54.6)	64 (15.5)	120 (29.1)	3 (0.7)	17.716, 0.606	**30.433, < 0.001**

**Table 12:** Perceptions of community beliefs regarding girls’ responsibilities and motivations for ATS

	Responses				Age group	Gender
Phrases	Most people or everyone	Half	Few or no one	No answer	Df = 12	Df = 3
How many people in your community believe that…	*n* (%)	*n* (%)	*n* (%)	*n* (%)	*x*^2^, *p-*value	*x*^2^, *p-*value
A girl with a developed body is ready to have sex	149 (36.2)	72 (17.5)	167 (40.5)	24 (5.8)	18.693, 0.096	2.793, 0.425
A girl with a developed body has the maturity to make decisions about her sexual partners and relations	136 (33.0)	75 (18.2)	175 (42.5)	26 (6.3)	17.856, 0.120	**12.533, 0.001**
A girl can refuse to get involved with a man that wants to be with her	230 (55.8)	78 (18.9)	85 (20.6)	19 (4.6)	18.355, 0.105	2.420, 0.490
If a girl responds positively to the advances of a man on the street, she should have sex with him	122 (29.6)	74 (18.0)	189 (45.9)	27 (6.6)	13.753, 0.317	3.973, 0.264
Girls that spend a lot of time in the street are available to get involved with men	187 (45.4)	73 (17.7)	136 (33.0)	16 (3.9)	15.883, 0.197	**12.675, 0.005**
Girls that use short skirts are looking for male attention	201 (48.8)	64 (15.5)	137 (33.3)	10 (2.4)	13.256, 0.351	**12.812, 0.005**
Girls that accept presents or protection from men should repay with sex	132 (32.0)	74 (18.0)	180(43.7)	26 (6.3)	12.889, 0.377	3.131, 0.372
Girls of 12 years old or younger are able to choose their sexual partners and relationships	58 (14.1)	49 (11.9)	281 (68.2)	22 (5.3)	9.722, 0.640	**13.865, 0.003**
Girls of 13 to 14 years old are able to choose their sexual partners and relationships	85 (20.6)	55 (13.3)	142 (34.5)	20 (4.9)	10.960, 0.532	4.702, 0.195
Girls of 15 years old or older are able to choose their sexual partners and relationships	179 (43.4)	98 (23.8)	119 (28.9)	16 (3.9)	19.652, 0.074	5.758, 0.124

Overall, the findings from the set of personal beliefs items and the set of social norms items were not closely related to each other. Gender was associated with nine items and age with six social norms items after logistic regression.

## Discussion

The quantitative data supported the qualitative finding that ADTS was normalized in these communities, with a quarter of men reporting that they had participated in transactional sex, 27.3% of the adult men reporting having had sex with minors, and about half of women reporting having had sex with adults while they were still minors. It should be noted that the prevalence of these behaviours may have been underestimated for multiple reasons, and thus may be higher than 27.3%. For example, survey questions measuring transactional sex may have failed to capture the actual prevalence of practice, particularly among men, since respondents can conflate transactional sex with sex work [27]. Additionally, in Brazil the age of consent is 14 years-old and sexual relationships with someone under 14 years can lead to an 8–15-year prison sentence. Thus, adults may be hesitant to report this practice despite assurances of confidentiality.

These findings suggest that ADTS was a common occurrence in these communities: almost half of respondents considered age-disparate relationships “normal” and expected their community members to think that it was normal too. When examining the reasons for ADTS, the items were related to girls’ responsibility, male desires and the benefits of ADTS. In the literature, the “sex for basic needs” paradigm tends to portray adolescents involved in ADTS as vulnerable victims [20], however, our findings highlight that community members view girls as the main agents responsible for ADTS, who seek these relationships not just to meet basic needs but to ascend socially. This is in line with the “girls as agents view” of transactional sex which has also been reported in past literature in sub-Saharan Africa [25].

The results also describe a lack of awareness of community, structural and social factors driving ADTS, and an over emphasis on individual risk factors, reinforcing the perception of ADTS as an issue pertaining to the private sphere.

When participants’ attributed responsibility for ADTS onto girls, adult figures who were notably missing from the discussion were the adult men engaging in ADTS, as well as fathers, teachers, health professionals and religious leaders. Mothers represented an adult female figure viewed as responsible for controlling or limiting the girls’ sexual behaviours. Unlike in Nkosana and Rosenthal [17] where girls autonomy and motives originate with the girls themselves, middle-aged participants and men in our study were more likely to attribute responsibility to the girls, maximize the benefits girls are reaping, and downplay the benefits men receive from participating in ADTS. Although adolescents were more likely to recognize the benefits that men receive from ADTS and their role as active participants in these relationships, adolescents also more clearly recognized the agency of girls, suggesting that the exclusive view of ADTS as exploitative may not hold true to this group either. It is important not to ignore the perceived benefits of these relationships for girls which may go beyond the material to also include emotional benefits [18, 25].

Although men were more likely to believe that a girl with a developed body is ready to have sex, they were not more likely to agree that a girl with a developed body has the maturity to make decisions about her sexual partners and relations. This contradiction portrays men’s views of girls’ bodies as passive, recipients of sex and not as people with agency. Simultaneously, men were also more likely to agree that girls manipulate their older, male partners. Men viewed girls as having multiple motivations for and gaining more benefits from engaging in ADTS: they believed that girls involved with men are seen as more mature and as more popular among other girls. Men also were more likely to believe that girls provide consent through indirect signals such as wearing short skirts, responding to advances of men on the street or accepting presents or protection from men. Thus, girls’ agency was recognized by men mostly when it served to victimize men or justify male behaviours [10].

Of the independent variables, gender was most frequently associated in multivariate analysis with personal belief items, followed by community and age (associated with 14, 9 and 9 items, respectively) (Table 10). Women in addition to men held expectations for themselves, younger girls and men that are based on gender norms and may support the belief that girls are responsible for ADTS [11]. Women, however, were less likely than men to place blame exclusively on girls. These findings show that both men and women need to question the social constructs of sexuality to end ADTS [9].

Strong religious beliefs were more frequently significantly associated with personal beliefs items, in some cases serving as a protective factor, than with social norms items. Participants with religious beliefs were less likely to have reported participating in age-disparate sex and were less likely to believe that girls aged 12 or younger were able to choose their sexual partners, or that a developed body signalled the maturity to make decisions about sexual partners. These participants were also more likely to believe that ADTS is less common when there are good public services. Although these points may remove some of the blame for ADTS from girls, this group supported the family as controllers of girls’ sexuality. Furthermore, the role of religion in protecting against ADTS is complex and should be further researched, especially in contexts like Brazil where there has been a strong push against sexual education and initiatives promoting gender equality for youth by religious communities [2].

In both the personal beliefs items and social norms, the men’s role in ADTS was minimized due to an overall acceptance of a notion of masculinity characterized by hypersexuality and lack of impulse control [9]. Despite the existence of a plurality of masculinities inter- and intra-societies, the concept of a hegemonic masculinity that reinforces rigid, traditional gender roles, where men possess domination over women and over men who do not portray the qualities of the hegemonic man, has been witnessed globally [11]. This hegemonic masculinity generally encompasses a set of key characteristics which include hypersexuality, limited displays of emotion and control over others [8].

Although not all participants believed in these characteristics of masculinity or expected most of their community members to believe in them, both the personal beliefs and social norms that promote ADTS were built upon concepts of hegemonic masculinity. It is important to identify and consider the characteristics of masculine identity in communities to design interventions because these gender norms influence both men and women. Additionally, public health interventions that do not problematize masculine role expectations in their behaviour change models can be limited in their ability to promote change and may even propagate harmful norms [7, 19].

The association of community of residence with 24 social norms items after multivariate analysis—more than any other variable—suggests that the social norms items were able to tease out community differences.

Current interventions focused on changing harmful practices are generally individualized and focused on the children and/or young women themselves to improve their ability to resist or denounce attempts at exploitative relationships [3, 7, 17, 18, 24]. Initiatives focused on preventing ADTS solely focused on girls reinforces the expectation that girls, despite their younger age and limited access to resources, bear the responsibility for ADTS, instead of sharing the responsibility with the older men who engage in ADTS. It is important to recognize girls’ as people with agency despite their age and gender, but extra care must be taken to not conflate agency with blame and to recognize the limitations on girls’ decision-making power [1]. This is particularly important in these study communities where girls face structural violence and limited education and employment opportunities, as well as social norms supporting adultism and patriarchy. The importance of acknowledging these types kinds of contextual issues when designing interventions is well described by Wamoyi et al. [26]:

“Finding the right balance between acknowledging this agency and contextualizing it within the structural constraints imposed on it is key if we want to go beyond principalist debates and really think about the best way of designing programs that help girls and young women live healthier lives and achieve their goals.” (p. 13)

Furthermore, the focus on girls as responsible for ADTS runs the risk of encountering another of common pitfall: overlooking the role of communities, social norms and structural conditions. More studies like this one which include the perspective of men when exploring ADTS are needed [13].

This study also identified other actors which serve as reference groups and play important roles in sanctioning or incentivizing ADTS: drug traffickers, friends and families. By including other actors in interventions, the dialogue on ADTS is nudged further out of the private sphere. In particular, the inclusion of drug traffickers in this context with high rates of gun violence and police conflict, speaks to the power dynamics in the community and their interplay with a culture of fear. The literature shows that machismo, or unequal gender norms, prevent bystander interference. Women’s interference in interpersonal or gender-based violence is especially limited by fear of becoming victims themselves. Especially in favela communities, there is a generalized culture of fear where talking too much, about the wrong topic or to the wrong person can have serious negative consequences [15, 21]. Drug traffickers, however, represent a source of symbolic, economic and physical power in the communities that were perceived to be able to overcome the “fear barrier”. It is interesting to note that the police were not cited in the qualitative phase as actors who could interfere in these relationships. In part the absence of government actors in the discourse of the residents of these communities stems from years of neglect forming historical and social constructs that distance the state from resident interests, despite the existence of legal frameworks for protecting youth [18].

Although the study has several strengths, including its mixed-methods design, it is not without limitations. Particularly, we could not include every factor identified in the qualitative study into the quantitative survey. Thus, qualitative findings that were more specific to the context and were perceived to have low generalizability were excluded. It should also be noted that the quantitative survey prioritized findings that were relevant to international experts with knowledge of a wide-range of international contexts.

## Conclusions

In this study, personal beliefs and social norms were not generally aligned, suggesting that initiatives working to change personal attitudes or beliefs regarding ADTS may not reflect changes in social norms even if changes in beliefs are reached. This study adds to the discussion on ADTS by considering both the personal beliefs and social norms of community members, including men, and supporting the growing body of evidence for interventions which go beyond the individuals directly engaging in these activities. Although the study was conducted in urban favelas in Rio de Janeiro, the hegemonic gender norms identified here have also been identified throughout the region and globally. Yet, our findings add to the literature by reinforcing the need to employ solutions which question the individualist character of ADTS, consider a wider range of actors as subjects and protagonists of interventions, and the collective forces acting in local and global contexts promoting or preventing ADTS. Additionally, these findings may support the protection of girls and adolescent women in child, early and forced marriages and unions. The findings highlight the importance of community-based education to target entrenched norms and values of males and females that support gender inequality and violence. They also point to the importance of involving men, boys and communities in violence prevention, e.g. mentoring, community engagement and bystander programmes, along with programmes expanding meaningful educational and professional opportunities for girls. Further research should consider the social norms around ADTS in different communities within and beyond the Global South, as well as validate an instrument for evaluating the impact of interventions on social norms.

## Data Availability

The datasets used during the current study are available from the corresponding author on reasonable request.

## Abbreviations

ADTS: Age-disparate transactional sex
B/CM: *Babilônia* And *Chapéu* *Mangueira*
CDD: *Cidade* *de Deus*
CM: *Complexo de* *Maré*
G/A: Girls and adolescents
NH: *Nova* *Holanda*
RO: *Rocinha*
SECA: Sexual exploitation of children and adolescents
SVI: Social Vulnerability Index
UPP: Pacifying Police Unit (acronym is for the Portuguese version)
